# Reasons for the low influenza vaccination rate among nurses in Slovenia

**DOI:** 10.1017/S1463423620000419

**Published:** 2020-09-30

**Authors:** Danica Rotar Pavlič, Alem Maksuti, Barbara Podnar, Mateja Kokalj Kokot

**Affiliations:** 1 Assistant Professor, Faculty of Health Sciences, Health Systems in the European Community, Managing and Improving Quality in Nursing, University of Primorska, Izola, Slovenia; 2 Institute for Political Management, Dunajska 106, 1000 Ljubljana, Slovenia; 3 Faculty of Medicine, University of Ljubljana, Ljubljana, Slovenia; 4 Department of Family Medicine, Faculty of Medicine, University of Ljubljana, Ljubljana, Slovenia

**Keywords:** nurse, influenza, vaccination rate, content analysis, qualitative study

## Abstract

**Aim:**

This study aimed to identify nurses’ views on influenza vaccination and factors that might explain why they do not receive influenza vaccinations, and to examine any ethical issues encountered in the vaccination process.

**Background:**

All 27 European Union member states and 2 other European countries recommended influenza vaccinations for healthcare workers in 2014–15. Data show that the influenza vaccination rate among nurses in Slovenia is even lower than in other European countries. Slovenian study showed that 41.7% of the respondents had received both the pandemic and the seasonal vaccine. Doctors had the highest level of vaccine coverage, with 44.1%, followed by registered nurses at 23.4%, whereas the lowest level was found among nursing assistants and nursing technicians (17%) at a Ljubljana health clinic.

**Methods:**

A qualitative study was carried out. Nineteen nurses who did not receive influenza vaccination took part in the study. Thematic interviews were conducted in December 2018. Interview transcripts were read, coded, reviewed and labelled by three independent researchers. The collected material was processed using qualitative content analysis.

**Findings:**

Thirteen categories and four themes were identified and coded, which enabled an understanding of the nurses’ views regarding influenza vaccination. Most of their experiences were positive in one way: they recognised the importance of vaccination and people’s awareness of it. However, they did not obtain the influenza vaccine themselves. The main barriers to vaccination were doubt regarding the vaccine’s effectiveness, the potential for side effects, the belief that young healthcare professionals are well protected and not at high risk, an overrated trust in their own immune systems, and the belief that pharmaceutical industry marketing was targeting them. The nurses suggested several ways that vaccination could be promoted and improved vaccination coverage achieved. These findings call attention to the importance of recognising both the need for targeted information for the nurses and the need for different approaches to healthcare provision.

## Introduction

Seasonal influenza is an acute respiratory infection caused by influenza viruses that circulate in all parts of the world. Most people recover from fever and other symptoms within a week without requiring medical attention. However, influenza can cause severe illness or death, especially in people at high risk. Worldwide, it is estimated that these annual epidemics result in about 3 to 5 million cases of severe illness, and about 290,000 to 650,000 deaths. Groups at greater risk than others include healthcare workers, due to their increased exposure to patients and risk of further spreading the disease, particularly to vulnerable individuals (WHO, 2018).

Healthcare workers (HCW) are an important priority group for influenza vaccination, not only to protect the individual and maintain healthcare services during influenza epidemics but also to reduce spread of influenza to vulnerable patient groups. Vaccination of HCWs should be considered part of a broader infection control policy for healthcare facilities (WHO, 2012). A meta-analysis of the incidence of influenza among HCWs and non-HCWs showed that HCWs have a significantly higher risk of influenza infection compared to employees outside this sector (Kuster *et al.*, 2011). The annual influenza attack rates range from 5 to 10% in adults (WHO, 2005); rates of 11%–59% have been reported in HCWs caring for patients with influenza (Salgado *et al.*, 2002).

All 27 European Union (EU) member states and 2 other European countries recommended influenza vaccinations for HCWs in 2014–15. In total, 24 of these had recommendations in place to vaccinate all HCWs; 4 recommended vaccination only for certain HCWs. In Northern Ireland and Scotland, influenza vaccine was offered to all HCWs, whereas England and Wales recommended that only HCWs in direct contact with patients should be vaccinated (European Centre for Disease Prevention and Control, 2017). HCWs’ knowledge, attitudes and behaviour regarding vaccination have significant impact on their patients’ decision-making processes (Nichol and Zimerman, 2001; Paget, 2009).

Surprisingly, although it would seem that those with medical knowledge should constitute a reasonable counterweight to this attitude, they are not much different from the general public. The HCWs one would expect to be most involved, not only because of their own health but also their responsibility to those they care for, are the least willing to obey. Those HCWs with especially poor vaccination coverage end up being those with the closest contact with patients: nurses (Pless *et al.*, 2017).

Slovenian regulations require the vaccination of HCWs that may be exposed to biological agents at their workplace or could infect other people. Vaccination-related issues in Slovenia are regulated by the EU Directive on the Protection of Workers from Risks Related to Exposure to Biological Agents at Work. This directive provides that employers must, according to their national laws, offer their employees free vaccination on the basis of a risk assessment of exposure to biological agents for which there is an effective vaccine. The National Institute of Public Health in Slovenia (NIJZ) encourages and promotes vaccination of HCWs. Nevertheless, vaccination coverage among Slovenian HCWs remains very low (Directive 2000/54/EC, 2000; Kraigher and Učakar, 2010; Petek and Kamnik-Jug, 2018). The first cross-sectional study among physicians and dentists in Slovenia to address their behaviour, knowledge and attitudes regarding pandemic and seasonal influenza vaccination was published in 2013. The study found that 41.7% of the respondents had received both the pandemic and seasonal vaccine (Sočan *et al.*, 2013). It found that doctors had the highest level of vaccine coverage, with 44.1%, followed by registered nurses at 23.4%, whereas the lowest level was found among nursing assistants and nursing technicians (17%) at a Ljubljana primary health centre (Hudnik, 2018).

The initiative for this study came from the primary healthcare practice of one of the authors. The 1918 influenza pandemic was the most severe pandemic in recent history. It affected around 500 million people and deprived of life as many as 50 million worldwide (Kirsty *et al.*, 2018). It was an unimaginable catastrophe and an enormous failure in an unfair battle, which affected the whole globe and made a permanent mark in history. During the 100th anniversary of the influenza pandemic, the author worked hard to vaccinate as many people on the patient list as possible. Nurses, on the other hand, did not want to obtain the influenza vaccine, and thus many patients were confused. This paper aims to sum up the reasons, beliefs and attitudes among non-vaccinated nurses and propose possible solutions to increase the vaccination rate in this professional group.

## Methods

First, a literature review was conducted. The following keywords were used in searches: *nurses, healthcare workers, vaccination, influenza.* Searches were performed using Boolean operators for PubMed: [(nurses) AND vaccination] AND influenza; [(healthcare workers) AND vaccination] AND influenza. The research included case reports, clinical trials, comparative studies, datasets, interviews, meta-analysis, reviews and systematic reviews written in English published between January 1998 and November 2018.The selection in PubMed was narrowed down to full-text, free or accessed through the University of Ljubljana Natl & Tech & Ctrl Libraries. The keywords selected had to be found in the article title or abstract, articles had to refer to nurses or HCWs including nurses and they had to be published in English.

The questionnaire was developed in the following manner: after studying the literature from Slovenia and abroad about vaccination among nurses, the first set of questions was developed. The researchers then discussed this set in order to narrow the topics. The exact wording of the questions was developed in consultation with a medical infectious disease specialist.

The next phase was dedicated to training the interviewers. Nine nursing students in the master’s programme at the University of Primorska, Faculty of Health Sciences, were trained in how to do qualitative research by interviewing. These trained interviewers conducted 19 interviews with nurses through purposive sampling. The following sampling criteria were applied: registered nurse, not vaccinated against influenza with written permission from the Head of the Department. There were no vaccination opponents among the participants. The sample covered all demographic groups: female and male, older and younger and from rural and urban areas. Interviewers were instructed how to select the interviewees in order to ensure the greatest possible diversity of the participants. A purposive sample size was determined on the basis of theoretical saturation, which is the point in the data collection process when new data no longer offer additional insights for the research question (Coyne, 1997). The saturation process was applied prospectively (Guest *et al.*, 2020), during the course of data collection, which allowed us to stop interviewing after a certain period of time. According to this condition, the final number of interviews conducted was 19. All of the interviews were audio-recorded and transcribed verbatim.

The interviews were used as a data collection tool. In accordance with the research goals and specifics of the target population, focussed interviews were conducted (Hopf, 2004; Johnson and Rowlands, 2014), for which it is characteristic that the theme of the discussion is known in advance and that data acquisition and data interpretation are carried out in an open manner. In addition to the planned pre-formulated questions, during the interviews we asked other sub-questions typical of focussed interviews. In this manner, we tried to provide the best conditions for the interviewees to have maximum opportunity to give the most extensive and detailed answers possible.

## Data analysis method

We conducted a qualitative study using qualitative content analysis. The key characteristic of this method is that extensive texts are classified into smaller content categories. The method contains an initial phase of preparation and organisation, including open coding, category formation and abstraction (Elo and Kyngäs, 2008; Saldaña, 2009; Schreier, 2012). After determining the coding units, we used open coding for the identification of categories and their classification (Elo and Kyngäs, 2008). First, we carried out a thematic analysis of the content. We started with the preparation phase, which included the selection of analysis units. In our notes and transcripts of the interviews, we marked those parts of the texts that we needed for further elaboration and that were connected with the research goals—that is, a sentence or more expressing a relevant declaration. In the qualitative synthesis, we used codes and categories, which mean that we classified the units with respect to their meaning (Saldaña, 2009). The lead author discussed the topics, categories and citations with the nine interviewers and recapitulated the reported interview content.

The study was approved by the National Medical Ethics Committee of Republic of Slovenia No 0120-522/2018/6. All interviewees gave written informed consent.

## Results

All the nurses who were invited agreed to participate. The nurses included in this study are professionals at all healthcare levels. The demography of the HCWs interviewed is presented in Table 1.

**Table 1.** Demography of nurses interviewed

Inter-view no.	Gender	Age	Level of care	Educational status	Years of work experience
1.1	F	32	Nursing home	Bachelor’s degree	10
1.2	F	57	Secondary healthcare	Bachelor’s degree	15
2.1	F	37	Primary healthcare	Secondary school	7
2.2	F	50	Primary healthcare	Bachelor’s degree	31
3.1	F	50	Tertiary healthcare	Bachelor’s degree	30
3.2	F	29	Tertiary healthcare	Bachelor’s degree	6
4.1	M	32	Primary healthcare	Secondary school	12
4.2	M	26	Secondary healthcare	Secondary school	3
5.1	F	43	Nursing home	Bachelor’s degree	20
5.2	F	45	Nursing home	Secondary school	24
5.3	F	33	Nursing home	Bachelor’s degree	10
6.1	F	32	Secondary healthcare	Bachelor’s degree	12
6.2	F	47	Tertiary healthcare	Bachelor’s degree	28
7.1	M	36	Tertiary healthcare	Not known	15.5
7.2	F	38	Tertiary healthcare	Bachelor’s degree	13
8.1	M	26	Tertiary healthcare	Bachelor’s degree	2
8.2	F	50	Tertiary healthcare	Bachelor’s degree	28
9.1	F	25	Tertiary healthcare	Bachelor’s degree	3
9.2	F	30	Tertiary healthcare	Bachelor’s degree	0.5

On the basis of the results obtained, the researchers formed 4 themes and 13 categories, in which 50 codes with a total frequency of 267 were identified. From these categories, we formed the following themes: 1) nurses’ experiences; 2) professionalism and protection procedures; 3) anti-vaccination beliefs and barriers and 4) types of vaccination promotion.

## Nurses’ experiences

The majority of nurses interviewed expressed the importance of disease severity and protection. Most of their experiences were positive, in a way: they recognised the importance of vaccination and people’s awareness of it. However, they did not obtain the influenza vaccine themselves. Some interviewees explained that they did not notice an increased presence of influenza, even in the home environment. Their main negative experiences were related to vulnerable subgroups of patients that are also most at risk. One such example is given here: ‘however, it is a completely different story for the elderly and patients with chronic illnesses, who are more affected by this infection and may be devastated by the flu’ (8.1).

The majority of nurses interviewed emphasised the importance of self-protection. They especially highlighted having a good immune system, a healthy lifestyle and healthy family members (ie, the domestic environment) and the importance of the health of entire population, free of epidemics and similar periods. Only three of the nurses interviewed emphasised the importance of a healthy working environment and the awareness that medical staff should get vaccinated. Here is one interesting example: ‘Yes, among the general population, in my personal experience, vaccination is relatively low, but most of my co-workers are vaccinated against the influenza every year at work’ (4.2).

## Professionalism and protecting procedures

Basically, all of the nurses interviewed explicitly stated that professionalism and protection procedures are important tools (or mechanisms) for greater vaccination. They particularly emphasised the importance of professionalism. The majority of nurses interviewed were absolutely convinced that health professionals are required to promote vaccination regardless of their personal beliefs. However, opinions were divided regarding how well they protect patients. Some nurses argued that non-vaccination does not endanger patients and family members. For example, nurse 1.2 said, ‘Although I have not been vaccinated, I have never been so sick as to endanger others’ (1.2).

Some of the nurses interviewed especially emphasised the importance of the protection of vulnerable groups (eg, the chronically ill) and compliance with safeguards. In this context, they listed disinfection, the use of protective masks and adherence to hygiene standards. For example, nurse 4.1 said, ‘To take precautionary measures and to protect them from transmission of infections from one patient to another’ (4.1).

Some of the nurses interviewed believe that chronically ill patients are at risk because of unvaccinated medical staff, and it is surprising that they still do not decide to be vaccinated. Some nurses equate influenza with other viral diseases; others do not have a reason for not being vaccinated. The obstacles to vaccination are, above all, doubts about the effectiveness of the vaccine and awareness that people sometimes become ill even though they have been vaccinated.

However, there is also an important emphasis on patient self-protection. Some of the nurses interviewed especially highlighted the importance of individual responsibility. One of the nurses interviewed was very clear: ‘it is an individual matter, and in the course of an epidemic every individual must know their rights and duties to act responsibly in these situations’ (9.2).

## Anti-vaccination beliefs and barriers

The main barriers to vaccination were doubt regarding the vaccine’s effectiveness, the potential for side effects, the belief that young HCWs are well protected and not at high risk, an overrated trust in their immune systems and the belief that pharmaceutical industry marketing was targeting them. Three categories were formed: doubts about the vaccine or belief in more effective measures, the impact of personal experience and the influence of the experiences of the local environment. The reasons against vaccination are shown in Table 2.

**Table 2.** The reasons nurses opted not to be vaccinated

Theme	Category	Codes	No of codes	Quote
Barriers	Doubts about the vaccine/belief in more effective measures	The vaccine is not effective enough	9	Low vaccination on the part of healthcare professionals, in my opinion, is due to awareness of poor vaccine efficacy. (6.2)
		Many side effects	7	I would also say that some have doubts about vaccine safety, but some also believe that there are more frequent reactions to vaccinations, which are almost more severe than the flu itself. (2.1)
		You get sick despite vaccinations	5	There is a general idea that these vaccines are not useful because those who have been vaccinated repeatedly say that they’ve had reactions or that they’ve gotten the flu anyway. (1.1)
		The vaccine only benefits certain patients	3	If I were a diabetic myself or had certain chronic illnesses. And really, in that case, the vaccine helps you not get so sick. In those cases. (1.2)
		No need because we are healthy	5	It seems to me that younger people don’t feel the need to get vaccinated because we feel healthy. (2.1)
		Safeguards are more important	1	Like me, because I’m young, if I don’t get the flu, if I’m generally well, vaccination is no longer essential. More of those other things. (1.1)
		Pharmaceutical companies are taking advantage of us	2	Pharmaceutical companies would benefit from our work and make profit and money. (4.1)
	Impact of personal experience	Non-vaccination of colleagues	1	I’ve been working for 10 years now and I’ve never been vaccinated and my colleagues aren’t vaccinated either. (1.1)
		I don’t get sick myself	3	I didn’t get the flu myself, because I never get sick. (4.2)
		Good immune system	4	I don’t believe that every disease requires vaccination, because the body itself develops certain antibodies necessary for human defence. I don’t have a specific reason for vaccination right now – I think I have a well-developed defence system. (8.2)
		Side effects after vaccination	2	When I got the pandemic flu vaccine years ago, I had a lot of side effects. Since then, I haven’t been vaccinated and haven’t become ill, which doesn’t mean that I won’t in the future. (2.2)
	Experiences of the local environment	Vaccinated people also fall ill	7	I’ve heard of many cases of people getting the flu vaccine and then having the flu that same year. (3.2)

## Types of vaccination promotion

**Figure 1.**
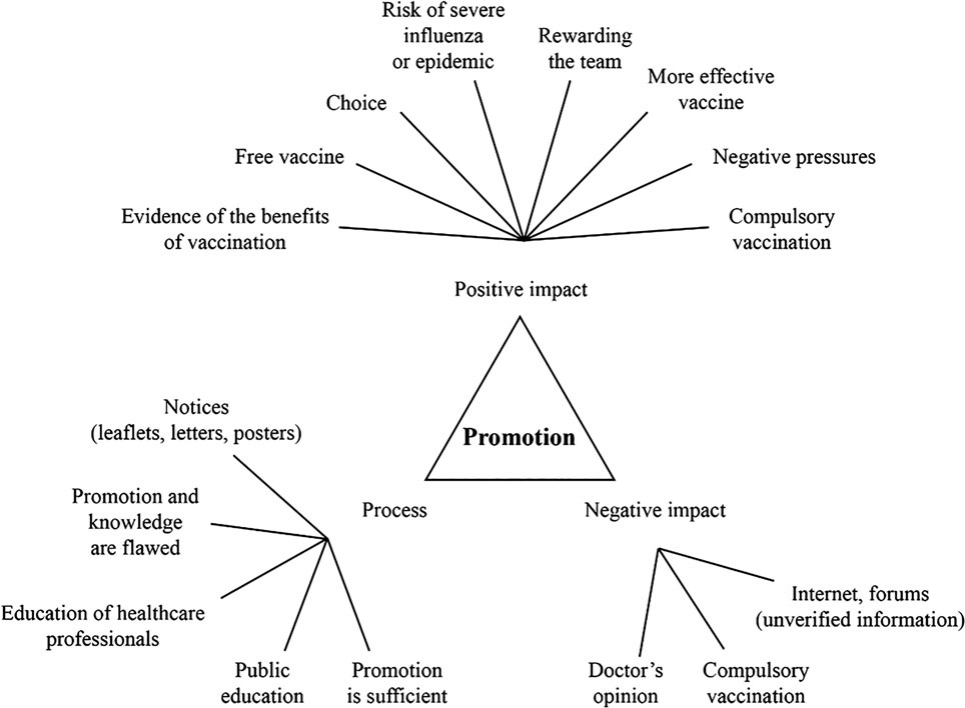
Approaches that could be used to promote influenza vaccination

Nurses suggested several ways in which vaccination could be promoted and increased vaccination achieved. Their suggestions were as follows: written notices (leaflets, letters and posters), targeted information for healthcare professionals, public education, free vaccines, autonomy and choice, risk of severe influenza or epidemic, rewards, internet forums (unverified information) and the importance of a doctor’s opinion. Figure 1 shows the approaches that could be used to promote influenza vaccination.

We looked at two groups of nurses: the first group consisted of nurses working at nursing homes or in primary healthcare, and the second group were those working in secondary or tertiary healthcare. We compared the answers from these two groups within the four identified themes.

Primary healthcare nurses more often did not recognise influenza as a common disease and more often stated that they had never had it themselves. Hospital nurses much more often expressed doubt that the vaccine was effective as a reason for not having been vaccinated. On the contrary, primary healthcare nurses more often expressed the conviction that the vaccination was not needed because they were in good health. Answers listing other reasons for not being vaccinated were relatively evenly distributed between the two groups. Both groups underlined the importance of promoting targeted information among HCWs, but primary healthcare nurses much more often stated that the existing vaccine promotion and the existing knowledge are inadequate both among HCWs and the general public.

Many hospital nurses stated that compulsory vaccination could have a positive impact on vaccination rates, but none of the primary healthcare nurses did. These much more often stated that vaccination free of charge or during pandemic danger could motivate people to seek vaccination, and that unverified information (from the internet, etc.) about disease and vaccination have a negative impact on vaccination rates.

## Discussion

Four themes (nurses’ experiences; professionalism and protection procedures; anti-vaccination beliefs and barriers and types of vaccination promotion) were identified in the decision-making by nurses that chose not to be vaccinated. The main finding of this study is the need for specific information targeted for HCWs. Other problem areas include familiar topics such as the notions that the vaccine is not effective enough, there are many side effects, medical staff can get sick despite vaccinations, there is no need because nurses are by nature healthy and have good immune systems, colleagues are non-vaccinated, vaccinated colleagues also fall ill, technical safeguards are more important and pharmaceutical companies are taking advantage of HCWs. Many argued that vaccines were ineffective. As a significant result, we also discovered the view that nurses would choose to be vaccinated if a severe epidemic or pandemic occurred (Lorenc *et al.*, 2017; Ozisik *et al.*, 2017). In one study, only 36.6% of the unvaccinated believed that available currently vaccinations are effective. In a Hong Kong study, 61% of nurses claimed that the vaccine could not protect them from illness (Asma *et al.*, 2016; Cheung *et al.*, 2017). Fear of side effects was the major and most frequently raised reason for vaccine refusal, even if the actual side effects reported by the participants themselves or their patients were rare and insignificant. A Chinese study revealed that 55.4% of nurses surveyed believed that vaccines may cause serious adverse effects (Zhang *et al.*, 2011; Lorenc *et al.*, 2017). The arguments about their own good health and immuno-resistance were common answers in other studies. Some stated that that they do not choose to be vaccinated because working in the hospital gives them the opportunity to train their own immune systems (Smith *et al.*, 2016; Lorenc *et al.*, 2017). It is obvious that nurses are constantly assessing the risk of infection and do choose vaccination in the event of major epidemics (Zhang *et al.*, 2012).

However, the tension between emphasising professionalism and scepticism about vaccination and its value in protecting at-risk groups and family members at home is surprising. When a nurse chooses to work in healthcare, she or he makes an autonomous choice to work in a service profession that serves the interests of vulnerable patients. Most nurses do not agree with mandatory vaccination, although some have mentioned rewards as a motivating factor. It would be reasonable for the national epidemiological service to decide whether a seasonal influenza vaccination of medical staff should be mandatory (Wicker and Marckmann, 2014.) By choosing not to be vaccinated themselves, nurses feed the fear of vaccines, reinforce anti-vaccination sentiment and set a bad example for their patients and the public. Mandatory influenza vaccination policies are in line with professional ethics. Nurses, however, experience the obligation to be vaccinated as an affront to their autonomy. Only one nurse indicated that she was influenced by a physician’s statement. Nurses did not mention a higher incidence of non-vaccination and absence from work. Furthermore, based on the literature, vaccination helps to maintain a stable workforce. An example is set that allows for fair involvement with others working in the hospital, as well as with the general public, to take the correct position on vaccination (Caplan, 2011). Our results differ from those of other studies, which revealed that nurses were more likely to accept vaccinations when considering them as a matter of professional responsibility (Smith *et al.*, 2016). We believe that non-vaccinated nurses in our study were also influenced by negative connotations in modern popular media and lack of educational interventions. It is not easy to explain why nurses are influenced by the media. This was an unexpected finding in our study. An Israeli study found that a media scare that occurred during the vaccination period influenced the decision not to get immunised in 34.1% of HCWs who did not choose immunisation (Abramson and Levi, 2008). A study from Turkey revealed that most HCWs were not willing to take up the vaccine during the H1N1 pandemic. Personnel that depended mainly on the media either did not accept vaccination or were undecided. The study authors concluded that social networks are also influential factors in the decision-making process. That is why it is important to empower HCWs through supporting the skills of acquiring and using evidence-based information (Hidiroglu *et al.*, 2010).

Studies from other countries indicate that risk perception was an exceptionally important factor: the more people know about the vaccine, the more risk they perceive but also the higher their vaccine uptake is (Zhang *et al.*, 2011; Wang *et al.*, 2015; Smith *et al.*, 2016).

Research in Croatia, France, Greece and Romania has investigated concerns HCWs might have about vaccination. The interviews did not specifically define the type of vaccination, but questions about hesitation applied to all vaccinations. The results revealed that vaccine hesitancy is present in all four countries among vaccine providers. The most important concern across all countries was the fear of vaccine side effects (Karafillakis *et al.*, 2016). Vaccination hesitation is also found in other types of vaccines, not just against influenza. New vaccines, such as the HPV vaccine, were singled out due to perceived lack of testing for vaccine safety and efficacy. This confirms previously conducted studies, which also showed HCWs’ concerns about new vaccines (Valour *et al.*, 2013). In this study, primary healthcare nurses more often did not recognise influenza as a common disease and more often stated that they had never had it themselves. The majority of nurses were aware that influenza is a serious illness for high-risk groups such as the elderly, people with chronic diseases and the immunocompromised (Toronto and Mullaney, 2010). A similar result was found in New Zealand, where practicing nurses agreed that influenza can be serious in older people (Brunton *et al.*, 2004). Slovenian primary healthcare nurses expressed the conviction that the vaccination was not needed because they were in good health. The interviews with Slovenian healthcare professionals showed that they value a healthy lifestyle as a defence mechanism against influenza (Petek and Kamnik-Jug, 2018). Hospital nurses much more often expressed doubt that the vaccine was effective as a reason for not having been vaccinated. Doubts about effectiveness of and indications for the vaccine were most frequently mentioned in a German university hospital study (Hagemeister *et al.*, 2018).

## Strategies for improving vaccine coverage among nurses

Non-vaccinated nurses’ views are highly variable and even contradictory in our study. We believe that this lack of professional harmonisation also affects the small proportion of influenza-vaccinated HCWs in Slovenia. However, most nurses agreed that more efforts are needed to promote vaccination and specific information targeted for nurses. They also stressed the need for rewards and vaccination that is free of charge. Switzerland implemented such approaches: vaccination was made free of charge and readily available. Their campaign included brochures, posters, reminders about vaccination hours or lectures by an infectious disease specialist. However, after five years of intervention the vaccination rate rose significantly, but only in the subgroup of physicians: from 34% to 63%. Among nurses, the rate remained low and nearly unchanged (17% versus 20%), which means this campaign, regardless of the effort put into it, was not well targeted to all HCWs (Friedl *et al.*, 2012).

More HCW-targeted programmes, which would provide factual information and focus on specific concerns surrounding vaccine effectiveness and side-effect risks, are needed (Rhudy *et al.*, 2010; Lorenc *et al.*, 2017; Ozisik *et al.*, 2017). One study suggested including questions about influenza prevention on licensing exams (Rhudy *et al.*, 2010).

## Methodological considerations

In addition to its several strengths, such as thematic interviewing, consistent use of qualitative content analysis this study also has some limitations. A general limitation of the study is related to epistemological criteria and validity in qualitative research (Whittemore *et al.*, 2001) Although qualitative studies provide a wealth of detail, large-scale representative quantitative surveys are needed to capture a large amount of data and shed more light on HCWs’ experiences with the vaccination against influenza. Another limitation is linked to the purposive sample used in the study. Our study focussed on nurses who have not received the influenza shot because of their views. Although the number of interviewees seems small, the richness of their testimonies has offered enough information to reach conclusions. In a similar survey, saturation was reached at an even smaller number of nurses interviewed (Rhudy *et al.*, 2010). The analysis findings were re-examined in a discussion with the nine interviewers, but these were not triangulated with the interviewees. We are aware that the qualitative approach has its limitations (Merkens, 2004) and know that an additional study with random participant selection and a quantitative approach would ideally complement our findings, putting the importance of these findings in perspective.

## Conclusion

Nurses that were not vaccinated against influenza expressed doubts about the vaccine in our study and believed that other measures were more effective. They emphasized personal experiences and that they had been healthy because of their good immune systems. As a significant result, we discovered the view that nurses would choose to be vaccinated if a severe epidemic or pandemic occurred. The tension between emphasising professionalism and skepticism about vaccination and its value in protecting at-risk groups and family members at home was surprising. Our results differ from those of other studies, which revealed that nurses were more likely to accept vaccinations when considering them as a matter of professional responsibility. However, most nurses agreed that more efforts were needed to promote vaccination and specific information targeted for nurses.
